# Molecular Profiling and Selective Pro-Apoptotic Activity of a Pruning-Derived *Lavandula dentata* Leaf-Surface Extract in Colorectal Cancer Cells

**DOI:** 10.3390/ijms27125535

**Published:** 2026-06-18

**Authors:** Shiva Pouramin Arabi, Marcello Scivicco, Valentina Parisi, Emanuele Rosa, Nunziatina De Tommasi, Ammar Bader, Vitale Del Vecchio, Nunzio Antonio Cacciola, Lorella Severino

**Affiliations:** 1 Department of Pharmacy, University of Salerno, 84084 Salerno, Italy; 2 Department of Veterinary Medicine and Animal Production, University of Naples Federico II, 80137 Naples, Italy; 3 Department of Pharmaceutical Sciences, Faculty of Pharmacy, Al-Ahliyya Amman University, Amman 19328, Jordan; 4 Department of Pharmaceutical Sciences, Faculty of Pharmacy, Umm Al-Qura University, Makkah 21955, Saudi Arabia; 5 Department of Experimental Medicine, University of Campania Luigi Vanvitelli, 80138 Naples, Italy; 6 Department of Life Sciences, Health and Health Professions, Link Campus University, 00165 Roma, Italy

**Keywords:** *Lavandula dentata*, leaf-surface extract, pruning residues, methoxylated flavonoids, mitochondria-mediated apoptosis, colon cancer cells

## Abstract

*Lavandula dentata* is a medicinal and aromatic plant rich in specialised secondary metabolites, but the biomedical potential of leaf-surface metabolites recovered from pruning biomass remains poorly investigated. In this study, a pruning-derived leaf-surface extract of *L. dentata* was obtained by brief acetone immersion followed by *n*-hexane partitioning. Its chemical profile was investigated by ultra-high-performance liquid chromatography coupled with high-resolution tandem mass spectrometry analysis combined with feature-based molecular networking, which revealed an enrichment in methoxylated flavonoids and pentacyclic triterpenes, including oleanane- and ursane-like derivatives. The biological activity of the extract was evaluated in HCT116 colorectal cancer cells, MDA-MB-231 triple-negative breast cancer cells, and HaCaT keratinocytes. After 24 h treatment, the extract selectively reduced HCT116 cell viability in a concentration-dependent manner, with an IC_50_ of 27.8 ± 1.049 μg/mL, whereas MDA-MB-231 and HaCaT cells were less sensitive. Mechanistic analyses in HCT116 cells showed increased early and late apoptotic populations, mitochondrial membrane depolarisation, and enhanced cleavage of caspase-9, caspase-3, and PARP. These findings indicate that a chemically profiled *L. dentata* leaf-surface extract selectively impairs colorectal cancer cell survival by activating mitochondria-mediated apoptosis. The study also supports the valorisation of pruning-derived aromatic plant biomass as a source of bioactive natural products with potential biomedical relevance.

## 1. Introduction

Cancer remains one of the leading causes of morbidity and mortality worldwide and continues to represent a major public health burden despite substantial advances in prevention, diagnosis, and treatment [1,2,3]. Conventional anticancer therapies often show limited selectivity for malignant cells and may be associated with dose-limiting toxicity and the development of resistance, thereby sustaining the search for new agents with improved efficacy and safety profiles [4,5]. In this context, plant molecules constitute a key reservoir of structurally diverse bioactive compounds that can modulate multiple cellular pathways implicated in carcinogenesis, tumour progression, and treatment resistance [6,7,8].

Among medicinal and aromatic plants, the genus *Lavandula* (Lamiaceae) has attracted growing interest because of its long-standing traditional use and its richness in terpenoid and phenolic constituents with documented biological activities [9,10,11]. *Lavandula dentata* L. (French lavender) is widely cultivated in Mediterranean and semi-arid regions as an ornamental and aromatic shrub, and its aerial parts have traditionally been used for the relief of gastrointestinal disturbances, headache, rheumatic pain, and symptoms associated with nervous tension [9,12]. Experimental studies on *Lavandula* species, including *L. dentata*, have reported antioxidant, antimicrobial, and anti-inflammatory properties for essential oils and plant extracts, thus reinforcing the pharmacological relevance of this taxon [9,10,13,14,15,16].

Phytochemical studies indicate that *L. dentata* produces essential oils rich in oxygenated monoterpenes, including 1,8-cineole, together with a range of other terpenoids and non-volatile phenolic compounds, such as phenolic acids and flavonoids [13,14,15,17,18,19]. This chemical profile is of particular interest in oncology because several terpenoid and phenolics have been associated with cytotoxic, pro-apoptotic, antiproliferative, and redox-modulating effects in tumour models. Preliminary in vitro evidence suggests that selected *L. dentata* preparations may exert cytotoxic activity against human cancer cell lines, supporting further investigation of this species as a source of bioactive candidates [19,20,21,22,23,24,25].

However, important gaps remain in the current evidence base. Most published studies on *L. dentata* have focused on crude extracts or essential oils obtained from whole organs or aerial parts, whereas the contribution of metabolites specifically associated with leaf surfaces and glandular trichomes remains comparatively underexplored [14,15,17,18]. This distinction may be biologically relevant because glandular trichomes are major sites of accumulation and secretion of specialised metabolites in Lamiaceae, and surface-oriented extraction may therefore yield preparations that differ substantially from conventional whole-tissue extracts in both composition and bioactivity [18,26]. In addition, although cytotoxic effects have been reported for some *L. dentata* preparations, the currently available evidence remains limited for defining their selectivity towards malignant versus non-transformed cells, since only a small number of studies appear to assess these effects through explicit parallel in vitro comparison models [19,20,25].

In parallel with the increasing demand for plant-derived ingredients in the pharmaceutical, nutraceutical, and cosmetic sectors, growing attention has been directed towards the sustainable valorisation of agricultural and horticultural by-products. In *Lavandula* cultivation, pruning is routinely performed to maintain plant architecture, revitalise plants, and stimulate regrowth and flowering, generating biomass that may represent an underexploited source of secondary metabolites [9,27]. From a bioprospecting perspective, converting pruning residues into chemically characterised extracts is an attractive strategy to combine waste valorisation with the discovery of bioactive molecules. This rationale is supported by recent work highlighting the resource-efficient exploitation of residues from medicinal and aromatic plants and by studies specifically investigating essential oils and by-products obtained from lavender pruning residues [28,29]. Moreover, targeting superficial tissues and glandular secretory structures may be particularly relevant in Lamiaceae as these compartments are enriched in specialised metabolites and contribute significantly to the phytochemical profile of *Lavandula* species [11,26].

On this basis, the present study aimed to valorise *L. dentata* pruning waste by producing a chemically profiled leaf-surface extract (LVD) and assessing its anticancer potential through a comparative in vitro approach. Specifically, the objectives were: (i) to characterise the chemical composition of LVD, with emphasis on metabolite classes plausibly related to biological activity; and (ii) to evaluate its cytotoxic effects in human tumour and non-tumour cell models (HCT116 colorectal carcinoma cells, MDA-MB-231 triple-negative breast cancer cells, and HaCaT immortalised keratinocytes) in order to obtain preliminary evidence of biological activity and differential sensitivity between tumour and non-tumour cell models.

## 2. Results

### 2.1. Ultra-High-Performance Liquid Chromatography-High-Resolution Mass Spectrometry (UHPLC-HR-MS) Analysis

**Figure 1:**
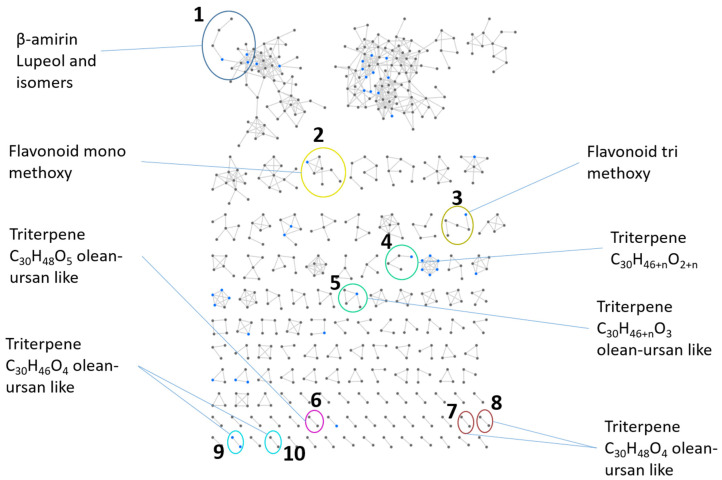
Feature-based molecular network of the pruning-derived leaf-surface extract of *Lavandula dentata* generated using GNPS2. Selected clusters putatively annotated as methoxylated flavonoids and pentacyclic triterpenes are highlighted. Numbered and circled regions identify the principal molecular families discussed in the text, including lupeol/β-amyrin-related nodes and triterpene clusters tentatively assigned as oleanan- and ursan-like derivatives.

**Figure 2:**
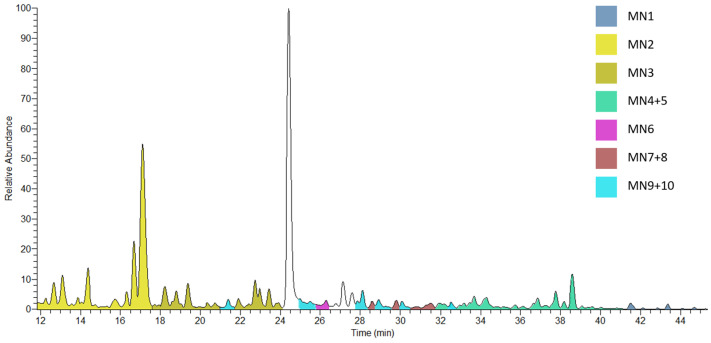
Ultra-high-performance liquid chromatography-high-resolution mass spectrometry chromatogram of the pruning-derived leaf-surface extract of *L. dentata*. Coloured peak regions correspond to the principal molecular networking clusters identified in Figure 1 (MN1, MN12, MN3, MN4+5, MN6, MN7+8, and MN9+10).

UHPLC-HR-Orbitrap/ESI-MS analyses and MN (Molecular Network) were performed on LVD. The results showed several clusters with compounds belonging to triterpene class, characterised by a C30 skeleton and molecular masses within the range of 425–489 g/mol (Figure 1). The tentative identification based on literature data was complicated due to the occurrence of several isomers and few literature information making it difficult to discriminate among already isolated molecules and compounds not yet described. On the other hand, MN strategy can help us to understand the complex mixture of extract that contain a large number of metabolites. Within the MN, each containing multiple nodes with closely related mass spectra were characterised by features indicative of triterpene structures, in terms of fragmentation patterns and predicted molecular formulae. In the MN2 cluster was present 7 parent ions at *m*/*z* between 285.0757 from 315.0862 attributable to mono-methoxylated flavonoids; these compounds showed loss at 15 Da corresponding to methyl group (-CH_3_). In the same way, MN3 cluster showed parent ion at *m*/*z* between 329.1015 from 345.0964 attributable to tri-methoxylated flavonoids, these ions showed 3 times the loss of 15 Da. These compounds were interesting for their proved biological activity and medicine application. The cluster MN4 indicated the presence of triterpene characterised by aldehyde group; MS2 shows typical loss of –CHO (29 Da), at *m*/*z* 441.3727. Unlike the MN5-6-7-8-9-10 clusters, which exhibit typical fragmentation patterns attributable to ursolic and oleanolic acid derivatives. The *m*/*z* values of these clusters range from 453 to 489 depending on the number of oxygen atoms linked to the C30 skeleton. They exhibit losses that are typical of hydroxyl (-(H_2_O)_n_) and carboxyl (CO_2_) groups. Furthermore, the presence of fragments at *m*/*z* 249 (C_16_H_25_O_2_^+^, directly originated from the RDA fragmentation of [M+H]^+^), 208 (C_14_H_24_O, directly originated from the RDA fragmentation of [M+H]^+^), 203 and 191 (respectively C_15_H_23_^+^ and C_14_H_23_^+^ were observed from the RDA fragmentation of [M+H−H_2_O]^+^ and [M+H−2H_2_O−CO]^+^) is indicative of the fragmentation of the C30 skeleton of ursolic and oleanolic acids, (Table 1) [30]. In particular, the fragments at 249 and 208 Da are diagnostic to elucidate the presence in C3 of hydroxyl group or carbonyl group in olean and ursan derivatives. On the other hand, MN1 presents mono-oxygenated triterpenoid, with a peak attributable to molecules such as β-amyrin and lupeol at *m*/*z* 427.3934. Figure 2 shows the peaks that correspond to the different clusters that were identified using GNPS2 (version 2026.05.21). These results suggest that the *L. dentata* extract contains large numbers of methoxylated flavonoids and triterpenes, which are known for their biological activities [31], as these clusters account for a large proportion of the peaks present in the chromatogram.

### 2.2. Differential Sensitivity of HCT116, MDA-MB-231, and HaCaT Cells to LVD

**Figure 3:**
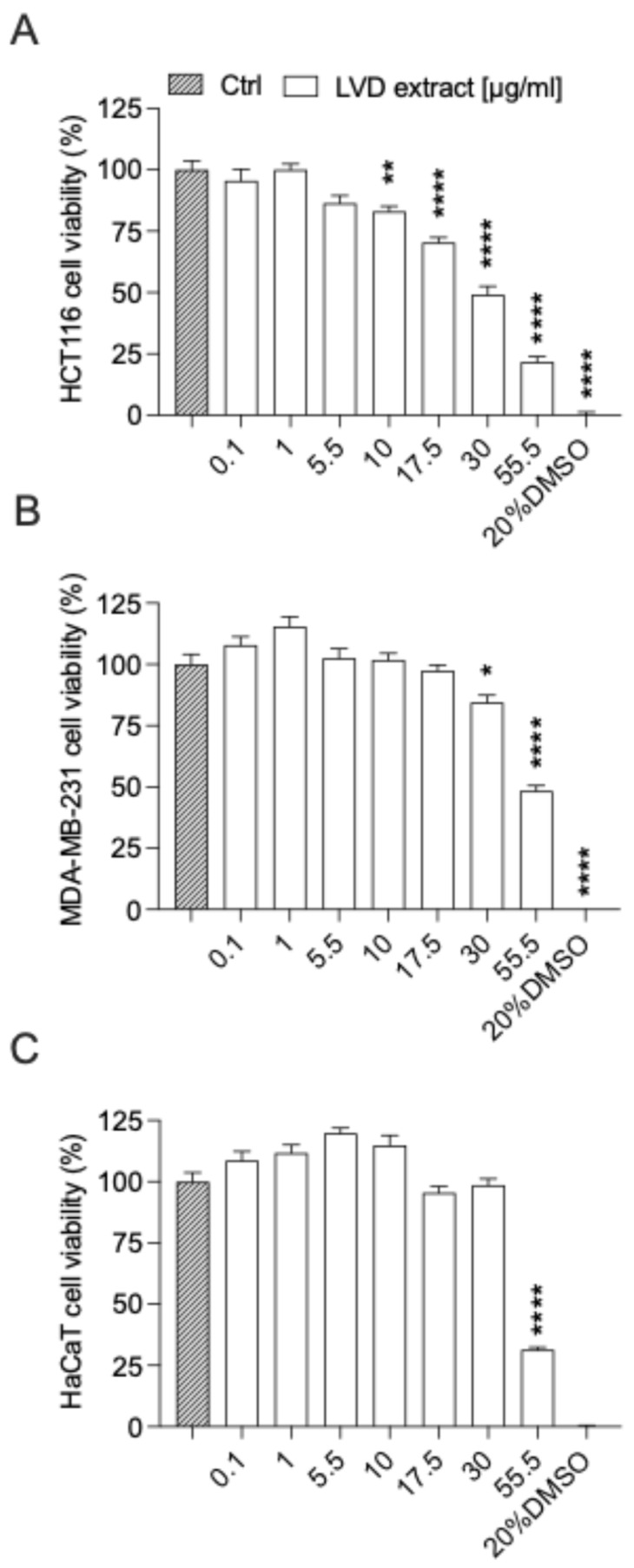
Effects of LVD on cell viability in HCT116, MDA-MB-231 and HaCaT cells. Cells were treated with increasing concentrations of LVD (0.1–55.5 μg/mL) for 24 h, and cell viability was evaluated by crystal violet staining. Viability is reported on a 0–100% scale, where vehicle-treated cells (Ctrl) were set at 100% and 20% DMSO was used as the full-kill control. (**A**) HCT116 cells displayed a marked concentration-dependent decrease in viability. (**B**) MDA-MB-231 cells were less sensitive to LVD, with significant effects detected only at higher concentrations. (**C**) HaCaT cells were the least sensitive, showing reduced viability only at the highest concentration tested. Data are expressed as mean ± SEM of three independent biological experiments, each performed with six technical replicates. Statistical analysis was performed using Brown–Forsythe and Welch ANOVA followed by Dunnett’s T3 multiple-comparison test. Statistical significance compared with control is indicated as * *p* < 0.05, ** *p* < 0.01, and **** *p* < 0.0001.

The effect of LVD on cell viability was evaluated in the human tumour cell lines HCT116 and MDA-MB-231, as well as in the non-tumour human keratinocyte cell line HaCaT, over the concentration range 0.1–55.5 μg/mL. Cells were exposed to the LVD extract for 24 h, and viability was assessed by crystal violet staining. As shown in Figure 3A, LVD induced a marked concentration-dependent reduction in HCT116 cell viability. No substantial effect was observed at 0.1 and 1 μg/mL, whereas viability progressively decreased from 5.5 μg/mL onward, with statistically significant reductions at 10 and 17.5 μg/mL. At 30 μg/mL, viability was reduced to 49%, and it further declined to about 21% at 55.5 μg/mL. Consistent with this response, the IC_50_ value for HCT116 cells was 27.8 ± 1.049 μg/mL. In contrast, MDA-MB-231 cells were less sensitive to LVD (Figure 3B). Cell viability remained close to control values up to 17.5 μg/mL, and a significant decrease was observed only at higher concentrations. At 30 μg/mL, viability remained around 84%, while at 55.5 μg/mL it decreased to 48%. Accordingly, the IC_50_ value for MDA-MB-231 cells was 54.1 ± 1.044 μg/mL. A similar concentration-dependent response was observed in HaCaT cells (Figure 3C). Viability was not significantly affected up to 30 μg/mL and remained close to control levels throughout this concentration range. At the highest tested concentration, 55.5 μg/mL, LVD induced a marked reduction in viability, to approximately 31%. Consistent with this observation, the IC_50_ value for HaCaT cells was 50.4 ± 1.057 μg/mL. Overall, these results indicate that HCT116 cells were the most sensitive to LVD treatment under the experimental conditions tested, whereas MDA-MB-231 and HaCaT cells showed lower and broadly comparable sensitivity. Based on these findings, HCT116 cells were selected for subsequent mechanistic studies, including Annexin V/PI apoptosis assay, JC-1 staining, and Western blotting.

### 2.3. LVD Induces Concentration-Dependent Morphological Changes in HCT116 Cells

**Figure 4:**
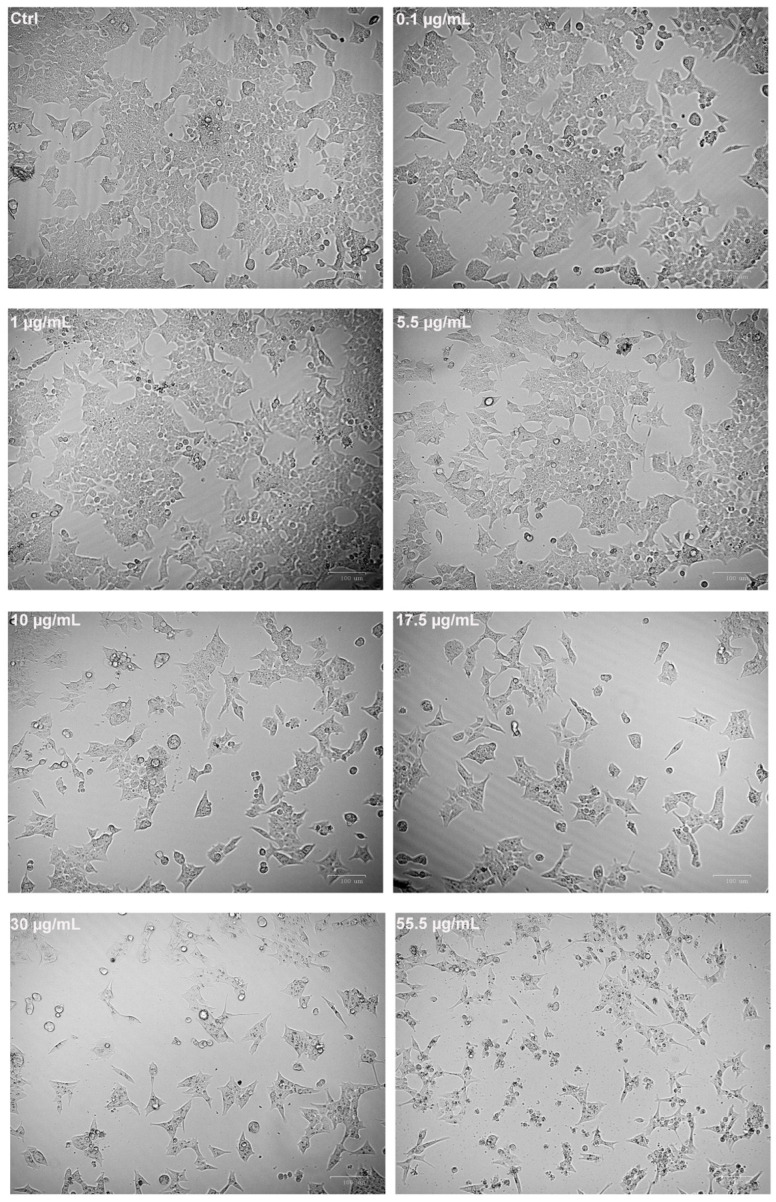
Concentration-dependent morphological changes induced by LVD in HCT116 cells. Representative phase-contrast images of HCT116 cells treated with increasing concentrations of LVD (0.1–55.5 µg/mL) are shown alongside untreated control cells (Ctrl). Control cells formed a dense, epithelial-like monolayer with preserved cell–cell contacts. LVD treatment caused progressive morphological changes, including reduced confluence, altered cell shape, increased cell rounding, partial loss of adhesion, and, at the highest concentrations, a marked reduction in adherent cell density with increased numbers of detached cells and cellular debris. Images are representative of independent experiments. Contrast and brightness were adjusted equally across all images for better visibility. Scale bars represent 100 µm.

Representative phase-contrast images showed that LVD induced concentration-dependent morphological changes in HCT116 cells (Figure 4). Untreated control cells displayed the typical morphology of adherent HCT116 cells, with a relatively dense epithelial-like monolayer and preserved cell–cell contacts. Cells exposed to the lowest concentrations of LVD, 0.1 and 1 µg/mL, appeared largely comparable to control cells, although a slight reduction in confluence and monolayer compactness was detectable. At 5.5 µg/mL, the monolayer appeared less compact, with reduced cell density and partial loss of intercellular contacts. These alterations became more evident at 10 and 17.5 µg/mL, where cells showed a clear decrease in confluence, increased cell rounding, and areas of partial detachment. At the highest concentrations tested, 30 and 55.5 µg/mL, LVD produced marked morphological alterations, characterised by a pronounced reduction in adherent cell density, disruption of the monolayer, increased numbers of rounded or detached cells, and the presence of cellular debris. Overall, these morphological changes are consistent with a concentration-dependent cytotoxic effect of LVD on HCT116 cells.

### 2.4. LVD Triggers Apoptotic Cell Death in HCT116 Cells

**Figure 5:**
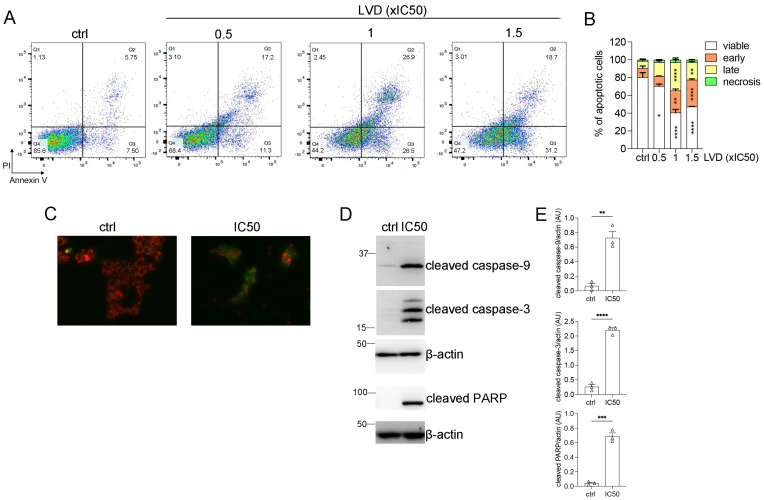
LVD induces apoptosis in HCT116 cells. (**A**) HCT116 cells were treated with LVD at 0.5 × IC_50_, 1 × IC_50_, and 1.5 × IC_50_ for 24 h. Apoptosis was then assessed by flow cytometry using Annexin V/PI staining. Representative dot plots are shown, and the percentage of cells is indicated in each quadrant: viable cells, lower left; necrotic cells, upper left; early apoptotic cells, lower right; and late apoptotic cells, upper right. (**B**) Quantitative analysis of flow-cytometry data showing the distribution of viable, early apoptotic, late apoptotic, and necrotic cells after treatment with LVD. Statistical analysis of Annexin V/PI data was performed by two-way ANOVA followed by Dunnett’s multiple-comparison test. (**C**) HCT116 cells were treated with LVD at the IC_50_ concentration for 24 h, and changes in mitochondrial membrane potential were evaluated by JC-1 staining. Representative images are shown. Red fluorescence indicates mitochondrial polarisation, whereas green fluorescence indicates depolarisation. (**D**) Cell lysates from HCT116 cells treated with LVD for 24 h were analysed by Western blotting (Figure S1) for cleaved caspase-9, cleaved caspase-3, and cleaved PARP expression. β-Actin was used as the loading control. (**E**) Densitometric analysis of the protein bands shown in panel D. Signal intensities were expressed in arbitrary units after normalisation to β-actin. Densitometric comparisons between treated and control cells were analysed using Student’s *t*-test. Data were obtained from three independent biological experiments. Statistical significance was defined as * *p* < 0.05, ** *p* < 0.01, *** *p* < 0.001, and **** *p* < 0.0001.

To determine whether the cytotoxic effect of LVD was associated with apoptosis induction, HCT116 cells were treated with increasing concentrations of LVD (0.5 × IC_50_, 1 × IC_50_, and 1.5 × IC_50_) for 24 h and analysed by flow cytometry using Annexin V/PI staining. As shown by the representative dot plots (Figure 5A) and the corresponding quantitative analysis (Figure 5B), LVD markedly reduced the viable cell population and increased the proportion of apoptotic cells compared with untreated controls. In control cells, the viable population accounted for 85.6%, whereas early apoptotic, late apoptotic, and necrotic cells represented 7.5%, 5.75%, and 1.13%, respectively. Treatment with 0.5 × IC_50_ LVD reduced the viable fraction to 68.4% and increased the early and late apoptotic populations to 11.3% and 17.2%, respectively, while necrotic cells remained limited (3.10%). Exposure to 1 × IC_50_ LVD produced a more pronounced effect, with viable cells decreasing to 44.2%, accompanied by a marked rise in both early apoptosis (26.5%) and late apoptosis (26.9%); necrosis remained low (2.45%). At 1.5 × IC_50_, the viable fraction remained markedly reduced (47.2%), while early apoptotic cells further increased to 31.2% and late apoptotic cells accounted for 18.7% of the total population; necrotic cells again represented only a minor fraction (3.01%). Overall, these findings indicate that LVD primarily induces apoptotic rather than necrotic cell death. When total apoptosis was calculated as the sum of early and late apoptotic cells, the overall apoptotic fraction increased from 13.25% in control cells to 28.5%, 53.4%, and 49.9% following treatment with 0.5 × IC_50_, 1 × IC_50_, and 1.5 × IC_50_ LVD, respectively (Figure 5B). To further investigate the involvement of mitochondrial dysfunction in LVD-induced apoptosis, mitochondrial membrane potential was evaluated by JC-1 staining. This analysis was performed qualitatively, based on the visual assessment of fluorescence signal distribution. As shown in Figure 5C, untreated cells exhibited predominantly red fluorescence, consistent with polarised and functionally intact mitochondria. By contrast, cells treated with LVD at the IC_50_ concentration showed a clear increase in green fluorescence together with a reduction in red signal, indicating mitochondrial membrane depolarisation. Consistent with these findings, Western blot analysis revealed a marked increase in the levels of cleaved caspase-9, cleaved caspase-3, and cleaved PARP in LVD-treated cells compared with control cells (Figure 5D). Densitometric quantification confirmed a significant increase in all three apoptotic markers following treatment (Figure 5E). Taken together, these results indicate that LVD triggers mitochondria-mediated apoptosis, leading to caspase activation and PARP cleavage.

## 3. Discussion

The present study demonstrates that the *n*-hexane-soluble leaf-surface extract of *Lavandula dentata* (LVD) exerts cytotoxic activity with differential sensitivity across the tested cell models, showing greater activity against HCT116 colorectal cancer cells than against MDA-MB-231 breast cancer cells and non-tumour HaCaT keratinocytes. This differential sensitivity represents one of the main findings of the study, since LVD reduced HCT116 viability in a clear concentration-dependent manner, with an IC_50_ of 27.8 ± 1.049 μg/mL after 24 h, whereas higher IC_50_ values were observed in MDA-MB-231 and HaCaT cells, namely 54.1 ± 1.044 μg/mL and 50.4 ± 1.057 μg/mL, respectively. Overall, these data indicate that LVD does not behave as a broadly non-specific toxic mixture but rather contains bioactive constituents with greater activity toward HCT116 colorectal tumour cells under the experimental conditions tested.

The phytochemical profile of LVD provides a basis for this biological activity. Ultra-high-performance liquid chromatography coupled with high-resolution tandem mass spectrometry (UHPLC–HRMS), combined with feature-based molecular networking, revealed that the extract is enriched in triterpenes and methoxylated flavonoids, including compounds tentatively assigned to lupeol- or β-amyrin-related skeleton, aldehyde-containing triterpenes, as well as several ursolic and oleanolic acid derivatives. These classes of natural products are known for their anti-proliferative and pro-apoptotic properties in several cancer models, including colorectal cancer. In particular, oleanolic acid has been reported to exert antiproliferative effects in HCT116 cells [33], while ursolic acid and its derivatives have also shown relevant activity in colorectal cancer models [34,35]. Likewise, lupeol has been described as an active anticancer compound in several colorectal cancer settings [36,37]. In addition, triterpene- and flavonoid-rich extracts from other *Lavandula* species, such as *L. stoechas*, have been associated with marked anticancer activity [38], further supporting the biological relevance of this phytochemical combination.

The greater sensitivity of HCT116 cells compared with MDA-MB-231 cells likely reflects differences in tumour type and intracellular signalling dependencies. HCT116 cells harbour oncogenic KRAS and retain wild-type p53, a molecular background known to favour apoptotic engagement under cytotoxic stress and discussed as a privileged therapeutic axis in colorectal cancer [39,40]. By contrast, MDA-MB-231 cells are representative of triple-negative breast cancer, a subtype characterised by high intrinsic resistance linked to strong activation of PI3K/AKT, MAPK, and NF-κB pro-survival pathways, as highlighted in recent reviews on triple-negative breast tumours and phyto-derived agents [41,42,43]. Although these mechanisms were not directly investigated here, the differential response observed in the present study is consistent with the distinct biological vulnerabilities of the two cell lines. Moreover, the higher sensitivity of HCT116 cells is in line with previous evidence showing that colorectal cancer cells are susceptible to triterpene-induced apoptosis mediated by ursolic acid, oleanolic acid, and related derivatives [33,34,35]. The limited impact of LVD on HaCaT cells up to 30 µg/mL further strengthens the relevance of the observed selectivity, suggesting a favourable in vitro therapeutic window, in agreement with studies that use HaCaT as a non-tumour reference line to define selectivity indices for natural compounds [44,45].

Morphological observations were fully consistent with the viability data. Phase-contrast microscopy showed a concentration-dependent transition from a dense, adherent epithelial-like monolayer to progressive loss of confluence, cell rounding, detachment, and accumulation of cellular debris in LVD-treated HCT116 cells. These alterations are consistent with the morphological features commonly associated with apoptosis induced by plant-derived cytotoxic agents. Similar changes have been described for *L. dentata* ethanolic extracts in MCF-7 cells, where cell shrinkage, rounding, and apoptotic bodies were observed together with DNA fragmentation and Annexin V positivity [20]. Comparable effects have also been reported for *L. angustifolia* essential oil in HNO210 laryngeal carcinoma cells, which showed reduced cell density, rounding, loss of intercellular contacts, and disruption of the monolayer [46], as well as for *L. angustifolia* extracts in HeLa and MCF-7 cells [25], for *L. stoechas* flower ethanolic extract in HT-29 colorectal cancer cells [47], and for triterpene- and polyphenol-rich preparations in other tumour models [37,48]. Therefore, the progressive accumulation of rounded and detached cells at higher LVD concentrations is in good agreement with the marked reduction in viability detected by crystal violet staining.

Mechanistically, our findings indicate that LVD induces apoptosis in HCT116 cells predominantly through a regulated cell death program rather than through primary necrosis. Annexin V/PI analysis revealed a concentration-dependent decrease in the viable cell population, accompanied by a parallel increase in both early and late apoptotic cells, while the necrotic fraction remained limited. These data support apoptosis as the main mechanism underlying the cytotoxic effect of LVD. Notably, although early apoptosis further increased at 1.5 × IC_50_, the proportion of late apoptotic cells was lower than that observed at 1 × IC_50_. This pattern may reflect differences in apoptotic kinetics at the 24 h time point, since Annexin V/PI analysis performed at a fixed endpoint captures the distribution of cells across different stages of apoptotic progression [49,50]. Consistent with this interpretation, total apoptosis remained markedly increased at both concentrations. This conclusion was further strengthened by JC-1 staining, which showed a marked loss of mitochondrial membrane potential, indicating mitochondrial depolarisation as an early event in the response to LVD. Since collapse of mitochondrial membrane potential (ΔΨ_m_) is a hallmark of intrinsic apoptosis and generally precedes caspase-9 activation and downstream executioner caspase signalling, these findings strongly support the involvement of a mitochondria-mediated apoptotic pathway [51,52,53,54]. Western blot analysis confirmed this scenario, showing that LVD markedly increased the levels of cleaved caspase-9, cleaved caspase-3, and cleaved PARP, thereby providing robust biochemical evidence of activation of the canonical intrinsic caspase cascade downstream of mitochondrial dysfunction.

Our results are also consistent with previous reports showing that *Lavandula*-derived preparations can promote caspase-dependent apoptosis in tumour cells. For example, lavandin essential oil induces dose-dependent Annexin V/PI-positive apoptosis and caspase-3 activation in HL-60 leukaemia cells, whereas the flower ethanolic extract of *L. stoechas* shifts HT-29 colorectal cancer cells from viability toward early and late apoptotic populations while increasing TP53 and CASP3 expression [47,55]. However, those studies did not systematically assess mitochondrial membrane potential. The present data therefore extend this body of evidence by demonstrating that a leaf-surface extract of *L. dentata*, enriched in triterpenes and methoxylated flavonoids, activates a mitochondria-mediated apoptotic programme in HCT116 cells. This response is characterised by the loss of mitochondrial membrane potential (ΔΨ_m_), together with the cleavage of caspase-9, caspase-3, and PARP. This mechanistic profile is also coherent with the phytochemical composition of LVD. Pentacyclic triterpenes such as oleanolic acid, ursolic acid, and lupeol have been widely reported to induce mitochondrial dysfunction and apoptosis in colorectal cancer cells through modulation of signalling pathways including Akt/GSK3β, Hippo, NF-κB, and Wnt/β-catenin [33,34,56]. The flavonoid fraction may also contribute to this effect, since methoxylated flavonoids have been associated with redox imbalance, mitochondrial perturbation, and caspase-dependent apoptosis in tumour cells [57,58]. In colorectal cancer models, flavonoid-induced cell death is frequently linked to ROS accumulation, mitochondrial dysfunction, and activation of apoptotic execution pathways [59]. Although ROS levels were not measured here, the coexistence of methoxylated flavonoids and triterpenoid constituents in LVD suggests a multitarget phytochemical system in which distinct metabolite classes converge on mitochondrial damage and irreversible apoptotic commitment. Overall, these findings indicate that the antitumour effect of LVD in HCT116 cells is best interpreted as the consequence of a chemically complex yet mechanistically coherent phytochemical matrix enriched in triterpenes and methoxylated flavonoids. Rather than producing non-specific toxicity, LVD appears to preferentially impair colorectal cancer cell survival by promoting mitochondrial depolarisation and activation of the intrinsic apoptotic machinery.

From a broader perspective, an additional strength of this study lies in its sustainability dimension. LVD was obtained from pruning-derived *L. dentata* biomass, supporting the growing interest in the valorisation of aromatic plant by-products as sources of high-value secondary metabolites. Recent studies on lavender and other Lamiaceae residues have shown that pruning-derived or post-distillation biomass can retain significant amounts of bioactive non-volatile compounds, particularly phenolics and other specialised metabolites [28,60]. In this context, the present work not only identifies a biologically active extract but also supports a circular-economy approach in which underutilised agricultural residues are converted into phytochemically characterised preparations with pharmacological potential.

Several limitations should, however, be acknowledged. First, phytochemical annotation of LVD remains tentative and is based on UHPLC-HR-MS fragmentation and molecular networking; isolation and full structural elucidation of the major constituents are still required, particularly for highly isomeric pentacyclic triterpenes, as highlighted by detailed mass spectrometry fragmentation studies [30,61,62]. Second, because the plant material was collected from a single geographical area and at a single time point, inter-batch, seasonal, and inter-year variability of the extract could not be assessed in the present study. Future work should therefore include independent collections from different harvest periods, years, and/or locations to evaluate the reproducibility of the phytochemical profile and biological activity of LVD. Third the study is restricted to in vitro conditions, and no information is yet available on pharmacokinetics, bioavailability, or antitumour efficacy in vivo, all of which are critical issues for lipophilic triterpenes such as ursolic and oleanolic acid derivatives [35]. Fourth, HaCaT cells were used as a non-malignant epithelial reference model; however, they do not represent a normal colorectal epithelial counterpart to HCT116 cells. Therefore, a direct assessment of tumour selectivity in the colorectal context will require future studies including non-transformed colon epithelial cells, enabling a more rigorous evaluation of selectivity and mechanistic responses.

These limitations also define clear future directions. Isolation and structural characterisation of the major triterpene and flavonoid constituents will be essential to identify the principal active components and to explore potential synergistic interactions within the extract. In addition, validation in appropriate animal models of colorectal cancer will be necessary to establish whether the selective pro-apoptotic activity observed in HCT116 cells translates into meaningful preclinical antitumour efficacy.

## 4. Materials and Methods

### 4.1. Plant Material

Flowering aerial parts of *L. dentata* were collected in February 2024 at Wadi Thee Ghazal, in the Taif region, Saudi Arabia (21.094367° N, 40.379097° E). The plant material was identified by one of the authors, A. Bader. A voucher specimen (SA-IT/2024-1) was deposited in the herbarium of the Laboratory of Pharmacognosy, Faculty of Pharmacy, Al-Ahliyya Amman University, Jordan.

### 4.2. Extraction

Fresh aerial parts of *L. dentata* (850 g) were immersed in acetone for 30 s, as previously described [63], yielding 6.5 g of extract. The leaf-surface extract was then partitioned with *n*-hexane to give an *n*-hexane-soluble fraction (LVD, 5.5 g) and an *n*-hexane-insoluble fraction (1.5 g).

### 4.3. Ultra-High-Performance Liquid Chromatography Coupled with High-Resolution Tandem Mass Spectrometry (UHPLC-HR-MS) Analysis

Chemical characterisation of the LVD extract was performed using an Ultimate 3000 UHPLC system (Thermo Fisher Scientific Inc., Bremen, Germany), coupled with an Orbitrap Q Exactive Plus mass spectrometer (Thermo Fisher Scientific Inc., Bremen, Germany). Before injection, the extract was dissolved in methanol and centrifuged at 4000 rpm for 5 min at 25 °C; the clarified supernatant was then injected at 10 mg/mL. Chromatographic separation was carried out on a Luna^®^ C18 column (150 × 2 mm, 3 µm, 100 Å; Phenomenex^®^, Castel Maggiore, Bologna, Italy). The mobile phase consisted of water with 0.1% *v*/*v* formic acid (phase A) and acetonitrile with 0.1% *v*/*v* formic acid (phase B). Elution started at 30% B and reached 100% B within 40 min, at a flow rate of 0.2 mL/min. Mass spectrometry data were acquired in both positive and negative ionisation modes, scanning from *m*/*z* 100 to 1500, using full-scan combined with data-dependent MS/MS acquisition.

### 4.4. Liquid Chromatography–Tandem Mass Spectrometry (LC-MS/MS) Data Preprocessing Parameters

Raw data files were first converted from .raw (Thermo Fisher Scientific) to .mzML format through MSConvert, part of the ProteoWizard package (version 3) (Palo Alto, CA, USA). All subsequent preprocessing steps were carried out in MZmine 4.9.14 [64]. Noise filtering during mass detection was applied using thresholds of 5 × 10^5^ counts (MS1) and 2 × 10^3^ counts (MS2). Chromatographic feature detection relied on the ADAP builder, with a minimum of 4 consecutive scans, intensity threshold of 5 × 10^5^, absolute minimum height of 1 × 10^5^, and 10 ppm *m*/*z* tolerance. Overlapping peaks were resolved with the local minimum resolver. The settings used were: chromatographic threshold 90%, minimum RT search range 0.05 min, minimum height 5 × 10^5^, peak top/edge ratio ≥ 1.5, duration between 0.0 and 1.35 min, and at least 5 scans per feature. MS2 spectra were paired to MS1 features within a 10 ppm precursor tolerance. ^13^C isotopes and redundant ions were removed, and only features carrying associated MS/MS spectra were retained for downstream analysis, following the approach described in a previous study [65]. Feature lists were exported as .mgf and .csv files using the “Export molecular networking files” function.

### 4.5. Molecular Network Analysis

Spectral data (.mgf) and metadata (.csv) were uploaded to GNPS2 (version 2026.05.21) and analysed through the Feature-Based Molecular Networking (FBMN) workflow. Networking was built by requiring a minimum cosine score of 0.70, at least 5 matched fragment peaks, and an *m*/*z* tolerance of 0.02 Da for both precursor and fragment ions. For spectral library matching, the cosine threshold was kept at 0.70, but the minimum number of matching peaks was raised to 6 to improve annotation reliability [66].

### 4.6. Cell Lines and Culture Conditions

HCT116 (ATCC-CCL-247) human colorectal carcinoma cells and MDA-MB-231 (ATCC HTB-26) human triple-negative breast cancer cells were purchased from LGC Standards (Sesto San Giovanni, Milan, Italy), while HaCaT human immortalised keratinocytes were kindly provided by Dr Irma Cioccia (University of Benevento, Benevento, Italy). HCT116 cells were cultured in McCoy’s 5A medium (Euroclone, Milan, Italy), whereas MDA-MB-231 and HaCaT cells were maintained in Dulbecco’s modified Eagle’s medium (DMEM; Thermo Fisher Scientific, Waltham, MA, USA). All culture media were supplemented with 10% foetal bovine serum (FBS; Thermo Fisher Scientific, Waltham, MA, USA), 2 mM L-glutamine (SIAL, Rome, Italy), 100 U/mL penicillin, and 100 μg/mL streptomycin (SIAL, Rome, Italy). The medium was renewed every 48 h. All cell lines were routinely screened for mycoplasma contamination. Experiments were performed using HCT116 cells between passages 16 and 26, MDA-MB-231 cells between passages 9 and 20, and HaCaT cells between passages 5 and 15.

### 4.7. Preparation of LVD Stock Solution

For in vitro experiments, LVD was dissolved in dimethyl sulfoxide (DMSO) to prepare a 40 mg/mL stock solution. To ensure maximal solubility, the solution was briefly sonicated in a water-bath sonicator for 10 min at 37 °C (LBS 1, model 616.1010.04; FALC Instruments S.r.l., Treviglio, Italy). Working concentrations were prepared by dilution in cell culture medium immediately before use. Vehicle-treated control cells received the same final concentration of DMSO as LVD-treated cells. In all experiments, the final DMSO content in culture medium did not exceed 0.1% (*v*/*v*).

### 4.8. Cell Viability and Morphological Analysis

Cell viability was assessed by crystal violet staining following the method of Feoktistova et al. [67], with minor modifications. HCT116, HaCaT, and MDA-MB-231 cells were seeded in 96-well plates at densities of 7 × 10^3^, 8 × 10^3^, and 8 × 10^3^ cells per well, respectively, and allowed to adhere for 24 h. Cells were then treated with increasing concentrations of LVD (0.1–55.5 μg/mL) in medium containing 1% FBS for 24 h. After treatment, the medium was discarded and the cells were gently rinsed with 1× PBS, then stained and fixed with 0.5% crystal violet solution for 20 min at room temperature. Excess dye was removed by washing the wells with tap water, after which the plates were air-dried overnight (approximately 16 h) and the bound stain was dissolved in methanol. Absorbance was measured at 570 nm using a microplate reader (model 680, Bio-Rad, Hercules, CA, USA). Results are expressed as percentage of cell viability (n = 3 independent experiments with 6 replicate wells per condition). Morphological alterations induced by LVD (0.1–55.5 μg/mL, 24 h) were also evaluated by bright-field and phase-contrast imaging using a Zoe™ Fluorescent Cell Imager (Bio-Rad, Hercules, CA, USA) equipped with a 20× objective.

### 4.9. Apoptosis Analysis

The induction of apoptosis was evaluated by flow cytometry using an Annexin V/propidium iodide (PI) staining assay as previously described [68]. Briefly, HCT116 cells were seeded in six-well plates at a density of 4 × 10^5^ cells/well and incubated for 24 h in the absence or presence of LVD at 0.5×, 1×, and 1.5× IC_50_ in medium containing 1% FBS. After treatment, cells were collected, washed twice with 1× PBS, and resuspended in Annexin V binding buffer. Samples were then stained with 2 μL Annexin V and 2 μL PI for 15 min at room temperature in the dark. Apoptotic cells were analysed using a BD FACSCanto II flow cytometer (BD Biosciences, San José, CA, USA), and data were processed with FlowJo V10 software (WilliamsonWay, Ashland, OR, USA).

### 4.10. Mitochondrial Membrane Potential

Changes in mitochondrial membrane potential were qualitatively assessed using the JC-1 fluorescent probe (MT09, Dojindo Molecular Technologies, Tokyo, Japan). This lipophilic cationic dye accumulates in mitochondria according to membrane polarisation: red fluorescence indicates mitochondrial JC-1 aggregates in polarised mitochondria, while green fluorescence indicates the monomeric form associated with membrane depolarisation. HCT116 cells were treated with LVD at the IC_50_ concentration for 24 h, then incubated with 5 μM JC-1 for 1 h at 37 °C in the dark. Fluorescence images were then collected with an EVOS FL Cell Imaging System (Thermo Scientific, Rockford, IL, USA).

### 4.11. Cell Lysate Preparation

HCT116 cells were seeded in 100 mm dishes at a density of 1.5 × 10^6^ cells per dish and allowed to attach for 24 h. The cells were then exposed to LVD at the IC_50_ concentration in medium containing 1% FBS for a further 24 h. Control cells were treated with equivalent amounts of DMSO, the vehicle in which LVD was dissolved. After treatment, the cells were washed twice with ice-cold 1× PBS, harvested by scraping, and lysed in RIPA buffer (Cell Signaling Technology, Danvers, MA, USA). Lysates were briefly sonicated for 15 s and then centrifuged at 12,000× *g* for 10 min at 4 °C. The resulting supernatants were collected, and total protein content was quantified using the BCA protein assay (Thermo Fisher Scientific, Waltham, MA, USA).

### 4.12. Western Blot Analysis

Equal amounts of protein (30 μg) were separated by SDS-PAGE on 8% or 15% polyacrylamide gels and transferred onto nitrocellulose membranes (Bio-Rad, Feldkirchen, Germany) using a Trans-Blot Turbo transfer system (Bio-Rad). After transfer, membranes were blocked for 1 h at room temperature in TBS-T (20 mM Tris-HCl (pH 7.6), 137 mM NaCl, and Tween 20) containing 5% non-fat dry milk. Membranes were then cut according to the expected molecular weight of the target proteins and incubated overnight at 4 °C under gentle agitation with the following primary antibodies: anti-cleaved caspase-3 (#9664, Cell Signaling Technology, Danvers, MA, USA; 1:1000), anti-cleaved caspase-9 (#20750, Cell Signaling Technology, Danvers, MA, USA; 1:1000), and anti-cleaved PARP (#5625, Cell Signaling Technology, Danvers, MA, USA; 1:1000). β-Actin (ab8226, Abcam, Cambridge, UK; 1:5000) was used as a loading control and incubated for 1 h at room temperature. After three 5 min washes in TBS-T, membranes were incubated for 1 h at room temperature with horseradish peroxidase-conjugated secondary antibodies (Cell Signaling Technology, Danvers, MA, USA; 1:2000). Immunoreactive bands were visualised using an enhanced chemiluminescence detection system, and images were acquired with a ChemiDoc Imaging System and Image Lab 6.0.1 software (Bio-Rad Laboratories, Milan, Italy). Band intensity was quantified using ImageJ software version 1.52n (Wayne Rasband, National Institutes of Health, Bethesda, MD, USA), normalised to the corresponding loading control, and expressed as arbitrary units (AU).

### 4.13. Statistical Analyses

Statistical analyses were performed using GraphPad Prism 10.3.1 (GraphPad Software, San Diego, CA, USA). Outliers were identified using the ROUT test. Differences between a single treatment group and the corresponding control were analysed using Student’s *t*-test. For cell viability/cytotoxicity assays, comparisons among multiple groups were performed using Brown–Forsythe and Welch ANOVA followed by Dunnett’s T3 multiple-comparison test, as homogeneity of variances was not assumed. Annexin V/PI flow-cytometry data were analysed by two-way ANOVA followed by Dunnett’s multiple-comparison test. For cell viability experiments, raw absorbance values were first corrected for background and then normalised to a 0–100% scale using plate-specific controls according to the following formula: % viability = (OD sample − OD 20% DMSO)/(OD vehicle − OD 20% DMSO) × 100. Vehicle-treated cells (culture medium containing the same final concentration of DMSO as that used in LVD-treated wells) were used as the negative control and set at 100% viability, whereas cells exposed to 20% DMSO were used as the full-kill control and set at 0% viability. The 20% DMSO condition was used only for data normalisation and was not included in dose–response curve fitting. Half-maximal inhibitory concentration (IC_50_) values were determined by non-linear regression analysis using the log(inhibitor) versus normalised response model with variable slope. Results are expressed as mean ± SEM of n independent experiments. A *p* value < 0.05 was considered statistically significant.

## 5. Conclusions

This study shows that pruning-derived *Lavandula dentata* (LVD) leaf-surface metabolites represent a chemically distinctive source of bioactive compounds with selective pro-apoptotic activity in colorectal cancer cells. The enrichment of LVD in methoxylated flavonoids and pentacyclic triterpenes, together with its preferential effect on HCT116 cells, supports the biological relevance of surface-associated metabolites from aromatic plant biomass. Overall, LVD emerges as a sustainable phytochemical matrix capable of impairing colorectal cancer cell survival through mitochondria-mediated apoptosis. These findings strengthen the value of pruning residues as underexploited resources for natural product-based anticancer research and provide a focused basis for the further development of *Lavandula*-derived bioactive agents.

## Figures and Tables

**Table 1:** Mass spectra data (positive ion mode) of compounds detected and tentatively identified in LVD extract.

Peak	Compound	Rt	[M+H+]^+^	MS2	MSI *
1	Dihydroxydimethoxyflavone	14.38	315.0858	300; 282; 255	2
2	Dihydroxymethoxyflavanone	16.32	287.0905	269; 245; 227; 167; 147	2
3	7-Methoxyapigenin	16.68	285.0752	270; 242; 167; 174	1
4	4′-Methoxyapigenin	17.09	285.0752	270; 242; 167; 174	1
5	Dihydroxytrimethoxyflavone	18.78	345.0961	330; 312; 281; 241; 213	2
6	Triterpene C_30_H_46_O_4_	21.91	471.3460	452; 434; 392; 287; 213; 179	3
7	4′-7-dimethoxyapigenin	22.95	299.0903	284; 256; 194; 167; 149	1
8	Triterpene C_30_H_48_O_4_	24.98	471.3460	452; 434; 392; 287; 213; 179	1
9	Tormentic acid	26.27	489.3574	471; 443; 407; 201; 189	1
10	Triterpene C_30_H_46_O_4_	28.06	471.3460	452; 434; 392; 287; 213; 179	3
11	Triterpene C_30_H_46_O_4_	29.11	471.3460	452; 434; 392; 287; 213; 179	3
12	Triterpene C_30_H_48_O_4_	29.43	473.3625	427; 409; 391; 357; 249; 208; 201; 189	3
13	Triterpene C_30_H_48_O_4_	31.51	473.3625	427; 409; 391; 357; 249; 208; 201; 189	3
14	3β-hydroxyoleanan-28-oic acid	31.84	459.3832	441; 423; 413; 395; 205	1
15	Triterpene C_30_H_48_O_4_	31.95	473.3625	427; 409; 391; 357; 249; 208; 201; 189	3
16	Triterpene C_30_H_46_O_3_	32.18	455.3533	437; 409; 259; 203; 289; 177	3
17	Triterpene C_30_H_46_O_3_	32.54	455.3533	437; 409; 259; 203; 289; 177	3
18	Triterpene C_30_H_48_O_2_	32.96	439.3561	393; 315; 297; 203; 191; 189	3
19	Triterpene C_30_H_48_O_2_	33.47	439.3561	393; 315; 297; 203; 191; 189	3
21	Oleanolic acid	33.44	457.3676	439; 411; 393; 235; 203; 1991	1
21	Triterpene C_30_H_48_O_2_	34.31	439.3561	393; 315; 297; 203; 191; 189	3
22	Ursolic acid	34.31	457.3676	439; 411; 393; 235; 203; 1991	1
23	Triterpene C_30_H_46_O_3_	36.87	455.3533	437; 409; 259; 203; 289; 177	3
24	Olean-12-en-20-al	37.35	525.3777	407; 217; 203; 191; 189	1
25	20,28-dihydroxylupan-3-one	39.52	459.3832	441; 423; 405; 219; 207	1
26	Lupeol	41.54	427.3934	409; 229; 191; 189	1

* MSI level of identification according to Sumner et al., 2007 [32].

## Data Availability

The data supporting the findings of this study are included within the article and are available from the corresponding author upon reasonable request.

## Supplementary Materials

The following supporting information can be downloaded at: https://www.mdpi.com/article/10.3390/ijms27125535/s1.
