# Supplementary material for: Molecular Profiling and Selective Pro-Apoptotic Activity of a Pruning-Derived Lavandula dentata Leaf-Surface Extract in Colorectal Cancer Cells
Source: Int J Mol Sci. 2026 Jun 18;27(12):5535. doi: 10.3390/ijms27125535 (PMC13299121; doi:10.3390/ijms27125535)

Full original blots used for Figure 5 D. Each blot membrane was cut based on the standard band positions and then incubated with the appropriate antibodies.

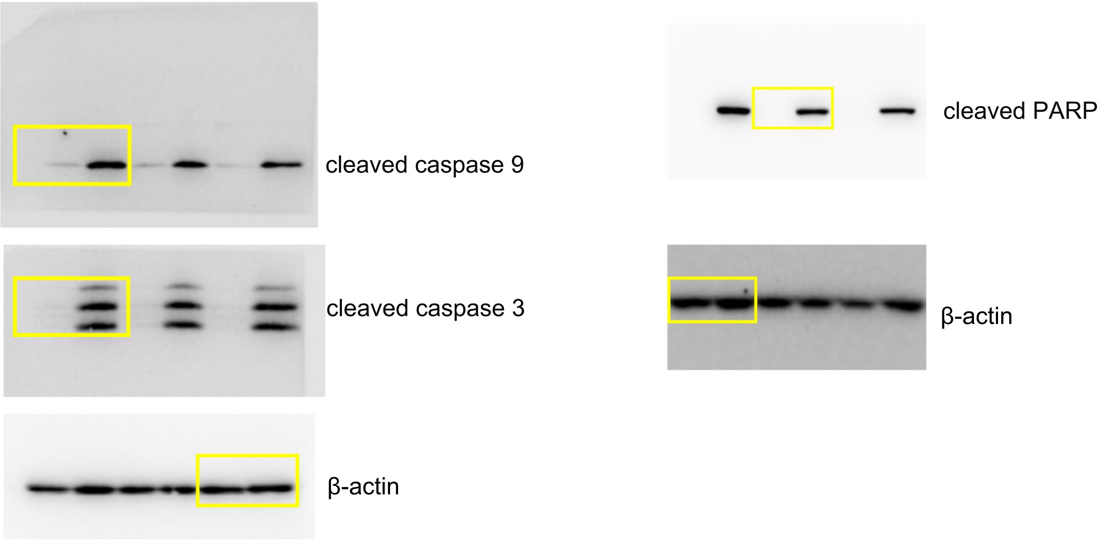

Supplement: Supplementary file 1 [file ijms-27-05535-s001.zip › ijms-4359807-supplementary.pdf]
